# Mild Temperature Thermal Treatments of Gold-Exfoliated Monolayer MoS_2_

**DOI:** 10.3390/nano15030160

**Published:** 2025-01-22

**Authors:** Emanuele Sangiorgi, Antonino Madonia, Gianmarco Laurella, Salvatore Ethan Panasci, Emanuela Schilirò, Filippo Giannazzo, Igor Píš, Federica Bondino, György Zoltán Radnóczi, Viktória Kovács-Kis, Béla Pécz, Gianpiero Buscarino, Franco Mario Gelardi, Marco Cannas, Simonpietro Agnello

**Affiliations:** 1 Department of Physics and Chemistry Emilio Segrè, University of Palermo, Via Archirafi 36, I-90123 Palermo, Italy; 2 Consiglio Nazionale delle Ricerche—Istituto per la Microelettronica e Microsistemi (CNR-IMM), Strada VIII, 5, Zona Industriale, I-95121 Catania, Italy; 3 Consiglio Nazionale delle Ricerche—Istituto Officina dei Materiali (CNR-IOM), Area Science Park, S.S. 14 Km. 163, 5, Basovizza, I-34149 Trieste, Italy; 4 HUN-REN Centre for Energy Research, Institute of Technical Physics and Materials Science, Konkoly-Thege ut 29-33, 1121 Budapest, Hungary; 5 ATEN Center, University of Palermo, Viale delle Scienze Ed. 18, I-90128 Palermo, Italy

**Keywords:** MoS_2_, gold-assisted exfoliation, 2D materials, Raman spectroscopy, photoluminescence, thermal processing, defects, strain, doping

## Abstract

Monolayer molybdenum disulfide is considered an extremely promising two-dimensional material for innovative electronics due to its direct bandgap and high charge-carrier mobility. The optical and electronic properties of monolayer MoS_2_ can, however, be strongly influenced by the specific synthesis route, posing challenges for industrial-scale production. In this study, we investigated the effects of moderate temperature thermal treatments under a controlled O_2_ atmosphere on the properties of monolayer MoS_2_ flakes. We found that the treatments can effectively tune the doping level of monolayer MoS_2_. Notably, 225 °C was identified as the optimal temperature for enhancing its optical emission properties. Our findings suggest that the removal of sulfur vacancies and impurities underlies these effects, demonstrating a promising approach for tuning the properties of monolayer MoS_2_ at mild temperatures.

## 1. Introduction

Since the discovery of graphene [1], interest in two-dimensional (2D) materials has grown steadily over the years due to their significant potential in several fields [2,3]. Nowadays, the many diverse 2D structures discovered and the promising properties they exhibit have allowed us to overcome the limits arising from the semimetal nature [4] of graphene in the context of application, especially in the case of devices based on semiconducting materials [5]. Among these, transition metal dichalcogenides (TMDs) are one of the most interesting classes of 2D materials with semiconducting properties, which have already shown their value for applications in electronics and optoelectronics [6]. Indeed, TMDs commonly present an intrinsic bandgap, which makes them suitable for semiconductor-based electronic devices such as field-effect transistors (FETs) [7,8] and light-emitting diodes (LEDs) [9]. All TMDs are characterized by a molecular layer structure with the general chemical formula MX_2_, where M is a transition metal (most commonly Mo and W) and X represents chalcogen atoms (most commonly S and Se). In the bulk structure of TMDs, atoms on a plane are held in place by strong covalent bonds, while different sheets are linked by van der Waals interactions. The weak out-of-plane bonds make it possible to reduce TMDs down to a 2D material composed of a single molecular layer, which often displays superior electronic and optical properties compared to its bulk counterpart.

Molybdenum disulfide (MoS_2_), and in particular, its monolayer structure (1L-MoS_2_), has attracted considerable attention as a 2D TMD material due to its abundance and ease of processing down to a single layer [10]. Bulk and monolayer forms of TMDs, as mentioned above, present significant differences in their properties. For instance, bulk MoS_2_ has an indirect bandgap of approximately 1.23 eV [11]. Due to the indirect nature of the transition occurring across the valence and conduction bands, no luminescence is exhibited by the bulk material. However, when MoS_2_ is reduced down to its monolayer form, a strong PL can be observed [12]. It has been demonstrated that in 1L-MoS_2_ the band configuration is modified compared to the bulk material, resulting in direct transitions with an energy separation of approximately 1.8 eV [13]. Specifically, while the valence band maximum in bulk MoS_2_ is located at the Γ point of the Brillouin zone, in 1L-MoS_2_ it shifts to the K point, where the minimum of the conduction band is also located [12]. This change has profound implications, as it enables 1L-MoS_2_ to exhibit emission driven by the direct radiative recombination of excitons. This transition is doubly resonant in energy due to spin-orbit coupling, which induces a splitting in the energy of the degenerate valence band [14]. The two components associated with the splitting are designed as the A and B exciton bands and are found at approximately 1.8 eV and 2.0 eV, respectively. Additional PL components are also observed due to interactions between excitons and other charge carriers. For instance, the A exciton is often deconvoluted into two different contributions, respectively, indicated as the A^0^ component, associated with neutral exciton recombination across the bandgap, and the A^−^ component, due to negatively charged trions [15,16].

The optical properties of 1L-MoS_2_ are sensitive to mechanical stimuli, an important feature reflecting this material’s potential for integration into bendable electronics. Experimental studies have shown that monolayer MoS_2_ flakes exhibit a very high in-plane Young’s modulus of 130 N m^−1^ and an intrinsic strength reaching its theoretical limit, more than 10% of the elastic modulus, and equal to 16.5 N m^−1^ [17]. Such properties are due to the strong covalent molecular bonds formed by the overlapping of the 4d Mo and 3p S electron orbitals [18]. This flexibility enables this material to withstand the application of significant mechanical stress, a feature that has been exploited to tune the electronic structure of 1L-MoS_2_ [19].

When considering the use of 1L-MoS_2_ in devices, its electronic properties are fundamental. Early studies of the charge mobility of monolayer flakes found values lower than 3 cm^2^ V^−1^ s^−1^, which can be considered insufficient for practical applications [20]. Subsequent research efforts have succeeded in pushing these values up to 200 cm^2^ V^−1^ s^−1^ by encapsulating the flakes in high-κ dielectrics such as HfO_2_ [21]. It has been demonstrated that both the electronic and optical properties of 1L-MoS_2_ are sensitive to the effect of dielectric screening [22] and that the substrate on which the samples are placed can significantly influence the observed properties [23]. At the same time, 1L-MoS_2_ characteristics are strongly affected by defects present in its molecular structure [24]. Sulfur vacancies (*V*_S_), a prevalent form of naturally occurring defects in MoS_2_ [25], introduce donor-like levels in the band structure of this material, thereby inducing its commonly observed n-type conductivity [26]. This property has been exploited through precise atomic-scale manipulation [27] or via molecular soldering [28] to modify the electronic structure of 1L-MoS_2_ and to obtain flakes tailored for specific applications.

Nowadays, various physical and chemical production techniques exist for 1L-MoS_2_, each suited to meet specific needs. An example is the mechanical exfoliation of bulk MoS_2_; because of the superior quality of the solid, crystalline precursor facilitates the production of monolayer flakes with very low defect density, thereby preserving key physical properties such as charge-carrier mobility [29]. Although substrates such as gold have been found to offer optimal adhesion with MoS_2_ [30], due to the mechanical nature of exfoliation, this technique generally limits production efficiency and significantly hampers the scalability of the process. Other common techniques such as chemical vapor deposition (CVD) follow instead a bottom-up approach, which enables the efficient growth of uniform flakes over large areas of a predefined substrate through chemical reactions [31]. The CVD process appears promising for the large-scale production of 1L-MoS_2_; however, despite its high scalability and suitability for industrial transfer, CVD-produced flakes typically exhibit a high defect density [32].

The electronic and optical properties of 1L-MoS_2_ can be extensively tuned through post-production techniques such as strain application, doping, or chemical processes. These approaches expand their application potential beyond the limitations imposed by the synthetic route. For example, plasma treatments carried out under a controlled atmosphere have shown a certain degree of control over the defectiveness of this 2D material [33,34]. Additionally, thermal annealing induced by laser pulses was demonstrated to be able to improve the crystallinity of 1L-MoS_2_ flakes [35]. Finally, thermal treatments have also been attempted on insulating substrates, although the process was often studied at temperatures higher than 300 °C, which could induce damage to the structure of the studied flakes [36,37].

Based on this perspective, we have examined the impact of thermal treatments carried out under a controlled O_2_ atmosphere on 1L-MoS_2_. In this work, we show how these effects can be exploited to enhance the properties of the flakes without causing any structural damage. To achieve this, we studied the properties of commonly available 1L-MoS_2_ exfoliated on gold using micro-Raman and micro-photoluminescence (PL) spectroscopy and demonstrated how these are influenced by the properties of the substrate itself. Subsequently, we investigated the effects of thermal treatments under a controlled atmosphere on the properties of the studied samples. Alterations in the structure of the studied material were observed down to the atomic scale through atomic force microscopy (AFM) and high-resolution transmission electron microscopy (HRTEM). This study, along with additional investigations using X-ray photoelectron spectroscopy (XPS), allowed us to identify the optimal treatment conditions to modify the electronic and structural properties of 1L-MoS_2_ while observing at the same time an overall enhancement in its optical emission. We consider these results useful from the point of view of an industrial transfer, where separating the production route from the final properties of the material enables the selection of a convenient synthetic process without sacrificing product quality. Finally, through an extensive comparison of treatments carried out under different conditions, we pinpointed the causes underlying the observed effects, helping to shine a light on the fundamental physics of 1L-MoS_2_ and on the origin of its enticing properties.

## 2. Materials and Methods

The 1L-MoS_2_ samples used in this experiment were prepared by gold-assisted exfoliation. Thin films of Ni (10 nm) and Au (either 2 nm or 10 nm) were sputtered on a SiO_2_ (900 nm)/Si substrate using a DC magnetron sputtering in a Q300T D PLUS system (Quorum Technologies, Laughton, United Kingdom), as described in previous publications [29]. During the sputtering, the pressure was maintained at a maximum of 10^−2^ mbar. The sputtered substrate was then pressed against a bulk MoS_2_ stamp obtained from a freshly cleaved 2H-MoS_2_ crystal. Such a procedure was carried out immediately after the Au was sputtered onto the substrate to prevent surface contamination and enhance the exfoliation efficiency.

All thermal treatments on 1L-MoS_2_ flakes were performed inside a water-cooled THMS600-PS cell (Linkam Scientific Instruments, Salfords, United Kingdom). After the samples were placed and sealed inside the cell, the internal head space of the system was filled with a gaseous atmosphere of 2 bar of O_2_. The temperature was then raised at a rate of 100 °C/min. When the target temperature (between 150 °C and 300 °C) was reached, the samples were kept under treatment for durations ranging from 30 min to 3 h. After completing the treatment, the system was kept sealed and allowed to naturally cool down unless stated otherwise. Once the sample reached room temperature (20 °C) it was characterized without being removed from the sealed thermal cell.

Micro-Raman and micro-PL spectra were recorded in order to study the electronic and optical properties of the observed 1L-MoS_2_ samples. A LabRam HR-Evolution Spectrometer system (HORIBA France SAS, Lyon, France) coupled to a confocal microscope (pinhole size set at 200 µm) equipped with a 100× and a 50× LWD objective was used to record both Raman and PL spectra using a laser excitation source with a wavelength of 532 nm. The laser power was reduced to 5% of its 100 mW maximum using an ND filter in order to avoid sample degradation during the measurements due to the focused laser beam; when observing the samples inside the Linkam cell, the laser power was increased 10 times in order to take into account the different excitation and collection geometry caused by the presence of the cell window. A grating with 1800 lines/mm was used in order to acquire Raman data, while PL spectra were acquired with a 600 lines/mm grating. All spectra were collected across a 10 × 10 µm^2^ or 15 × 15 µm^2^ area as indicated in the text, with a step size of 1 µm for Raman measurements and 1.5 µm for PL measurements; values calculated from the Raman and PL spectra are reported as averages of all the different measurements acquired across the relevant areas. The Si Raman peak at 520.70 cm^−1^ arising from the samples’ substrate was used as a reference for the Raman spectral calibration to take into account eventual instrumental shifts. When considering the PL intensity, the integrated Raman bands of 1L-MoS_2_ have been used as references for normalization to take into account eventual excitation instabilities and focus position differences. Raman bands were fitted with Lorentzian-shaped peaks, while PL bands were fitted with Gaussian-shaped peaks. The reported uncertainties associated with quantities derived from curve fits correspond to three times the standard error obtained from the least-squares fitting procedure. When reporting average quantities, the corresponding associated uncertainties are equal to the standard error of the mean.

AFM micrographs were collected in air with a Dimension FAST SCAN microscope (Bruker, Milano, Italy) using the soft tapping mode and a FAST-SCAN-A probe (Bruker AFM Probes, Camarillo, CA, USA) (a 27 μm long triangular Silicon Nitride cantilever). The probe presents a nominal resonant frequency of 1400 kHz, a force constant of 17 N m^−1^, and a tip radius of about 5 nm. The AFM images were acquired across a 5 × 5 µm^2^ area.

To study the structural properties of 1L-MoS_2_ before and after the thermal treatments, a cross-sectional study was performed by transmission electron microscopy, using a Themis 200 G3 transmission electron microscope (Thermo Fisher Scientific, Eindhoven, The Netherlands) with a C_s_-corrected objective lens in HRTEM mode. Thin cross-sections of the flakes were cut by a focused Ga^+^ ion beam (FIB, Scios 2 dual beam, Thermo Fisher Scientific, Eindhoven, The Netherlands) with 30 kV beam energy for cutting and 5 kV beam energy for polishing. To protect the structure during preparation, a thin (~50–100 nm) carbon layer and a thick (~1.5–2 µm) Pt layer were deposited locally where the lamella was to be cut. Moreover, the carbon layer provided enhanced contrast, enabling high-quality imaging of the adjacent MoS_2_ layer. After the FIB preparation, all samples were cleaned by a low-energy Ar^+^ ion beam using a GentleMill (Technoorg-Linda Ltd., Budapest, Hungary) operated at 500 V and 300 V beam energies. This way, very thin samples were obtained with surfaces free from damaged/amorphized layers.

XPS measurements were carried out at room temperature in an ultra-high vacuum at the BACH beamline of the Elettra synchrotron facility (Trieste, Italy). The beamline is equipped with a hemispherical electron analyzer R3000 (Scienta Omicron, Uppsala, Sweden). The beam size on the sample was approximately 500 × 300 μm^2^. The spectra were collected at a take-off angle of 90° using a photon energy of 607 eV. The total energy resolution was set to 0.25 eV. Binding energies were referenced against a Au 4f_7/2_ (84.0 eV) signal from the Au substrate. Voigt line shapes and a Shirley-type background were used to fit the Mo 3d photoemission spectra.

## 3. Results

**Figure 1:**
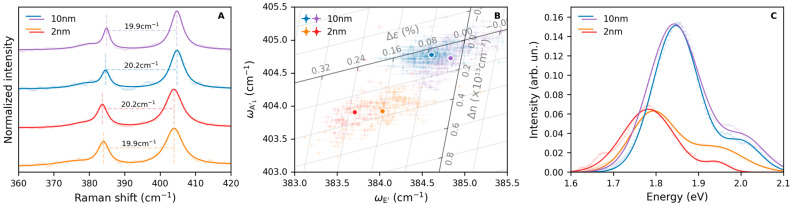
Raman spectra (**A**), strain-doping map (**B**), and PL spectra (**C**) recorded at different positions of pristine 1L-MoS_2_ flakes exfoliated on two different Au substrates of thickness equal to either 2 nm or 10 nm. Points in panels (**A**,**C**) represent the experimental data, and continuous lines represent the fits with Lorentzian (**A**) or Gaussian (**C**) bands (see text). The strain-doping map in panel (**B**) displays the points calculated across the surface of the studied flakes as partially transparent, while their average is shown as a solid color.

The morphological and spectral properties of pristine MoS_2_ flakes transferred on a gold substrate were studied by micro-Raman and micro-PL spectroscopy. The Raman spectra of the flakes exhibit the well-known E’ and A_1_’ bands, corresponding to the 1L-MoS_2_ in-plane and out-of-plane vibrations; these are located approximately at 385 cm^−1^ and 405 cm^−1^, respectively (Figure 1A). The Raman spectra were fitted with three Lorentzian-shaped peaks associated with the LO(M), E’, and A_1_’ modes. The peak-to-peak distance between the latter two bands, shown in the plot and approximately equal to 20 cm^−1^, matches the expected value for monolayer flakes [38]. The thickness of the sample was further analyzed using AFM, which confirmed that the observed flakes are monolayers with a thickness of 0.7 nm (Figure S1). In addition to the E’ and A_1_’ vibrational bands, a low-intensity in-plane longitudinal optical mode, LO(M), is observed in the Raman spectra peaking at approximately 377 cm^−1^ (Figure S2).

From the position of the Raman peaks, the strain and doping values of the studied flakes can be calculated [39]. In order to do so, the peak positions were evaluated through a Lorentzian least-squares fitting procedure, and the results are reported in Figure 1B. The strain and doping values were obtained in comparison to a reference undoped and unstrained sample, as described in the literature and detailed in the Supplementary Materials [39]. This reference sample was assumed to display the E’ and A_1_’ peaks, respectively, at 385 cm^−1^ and 405 cm^−1^ [40]. Following this method, we determined that the flakes deposited on both 2 nm and 10 nm Au substrates display n-type doping with charge-carrier concentrations of (0.339 ± 0.005) × 10^13^ cm^−2^ and (0.074 ± 0.005) × 10^13^ cm^−2^, respectively. At the same time, the flakes displayed a tensile strain equal to (0.194 ± 0.003)% and (0.048 ± 0.003)%, respectively. The strain-doping map shown in Figure 1B indicates that flakes deposited on the thicker 10 nm Au substrate are closer to the reference free-standing sample. In contrast, flakes deposited on the thinner 2 nm Au substrate present both higher strain and doping. The spatial distribution of strain and doping across the samples, obtained from Raman spectra maps acquired across a 10 × 10 µm^2^ square area with a 1 µm step (Figure S3), reveals relatively homogeneous properties across the flakes’ surfaces. However, small variations can be observed near the flakes’ edges, where both strain and doping levels are slightly lower compared to the interior regions of the sample.

The PL spectra of the samples are shown in Figure 1C. Each acquired PL spectrum displays two main bands fitted with Gaussian-shaped peaks. In the case of the 10 nm Au substrate, these were found at 1.85 eV and 2.0 eV (1.80 eV and 1.95 eV for the 2 nm Au substrate, Figure S4A,B) and have been associated with the A and B exciton emissions, respectively. Additionally, the PL of 1L-MoS_2_ flakes deposited on the thicker 10 nm Au substrate exhibit a higher blueshift compared to that of flakes deposited on the 2 nm thick gold substrate. Moreover, as shown in Figure 1C and Figure S3C, the flakes on the 10 nm gold substrate also present a higher PL intensity. Such properties make these samples more appealing from an applicative standpoint.

**Figure 2:**
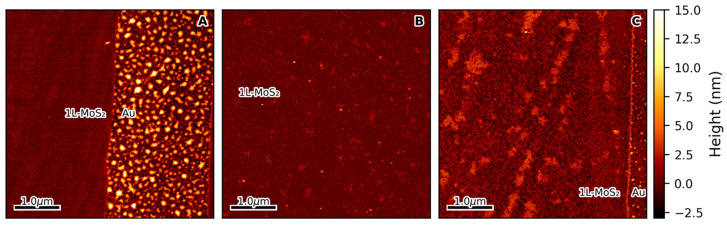
5 × 5 µm^2^ AFM morphology of a 1L-MoS_2_ flake exfoliated on a 10 nm thick Au substrate. The panels show the morphology of a sample in pristine conditions (**A**), after a 2 h thermal treatment carried out at 225 °C under a 2 bar O_2_ atmosphere (**B**), and after the same treatment was carried out at 300 °C (**C**). Regions associated with 1L-MoS_2_ and Au have been labeled accordingly.

Preliminary thermal treatments were performed at temperatures ranging from 150 °C to 300 °C. Samples undergoing these treatments were maintained at the target temperature for 2 h under a 2 bar O_2_ atmosphere. Molecular O_2_ was chosen for these treatments, as it has been reported that it can fill the sulfur vacancies (*V*_S_) inherently present in MoS_2_ [41] that are known to introduce n-type doping. As shown in Figure S5, optical microscopy images of the treated flakes acquired at a 10× magnification indicate the formation of extended structural defects as the treatment temperature is increased up to and above 250 °C, as evidenced by the different image contrast. These observations are corroborated by the morphology of the samples observed both by AFM and HRTEM (Figure 2 and Figure S6, respectively) and by AFM phase images (Figure S7). In particular, the surface of the pristine 1L-MoS_2_ flakes (Figure 2A and Figures S6A and S7A) does not display any clear structural change for treatments up to 225 °C (Figure 2B and Figures S6B and S7B), while it was strongly damaged after performing the 300 °C thermal treatment (Figure 2C and Figures S6C and S7C).

**Figure 3:**
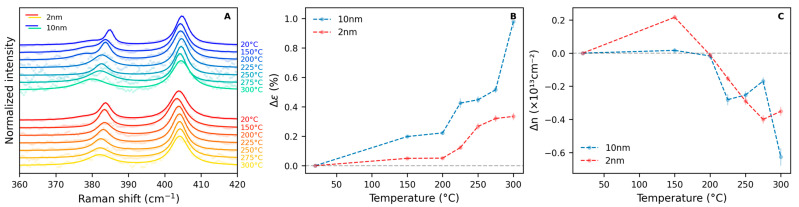
Raman spectra (**A**), strain variation Δε (**B**), and negative charge-carrier concentration variation Δn (**C**) of 1L-MoS_2_ flakes exfoliated on two different Au substrates of thickness equal to either 2 nm or 10 nm as a function of thermal treatment temperature. Points in panel (**A**) represent the experimental data, and the continuous lines represent the fits with Lorentzian bands.

Raman spectroscopy can provide valuable insight into the processes occurring during the thermal treatments. Representative spectra of the treated flakes (Figure 3A) show that, as the temperature is increased, the E’ band exhibits a progressive redshift, indicative of the accumulation of tensile strain within the flakes. Concurrently, following treatments above 225 °C, the full width at half maximum (FWHM) of the spectra starts to increase (Figure 3A and Figure S8), evidencing the degradation previously observed in the optical microscopy images. From the shifts in the position of the E’ and A_1_’ bands, it is possible to calculate the variations in strain and doping of the treated flakes as a function of temperature, as shown in Figure 3B and Figure 3C, respectively. The obtained strain curve displays a progressive increase in tensile strain, especially occurring at temperatures above 225 °C. In particular, in the case of 1L-MoS_2_ flakes deposited on the thicker 10 nm gold substrate, a net (0.98 ± 0.04)% increase in strain was observed after the 300 °C thermal treatment. At the same time, starting from 225 °C, the negative charge-carrier concentration of the samples progressively declines with the temperature of the treatments, reaching (0.63 ± 0.05) × 10^13^ cm^−2^ for the flake deposited on the thicker 10 nm gold substrate and (0.35 ± 0.03) × 10^13^ cm^−2^ for the flake on the thinner 2 nm gold layer after the 300 °C thermal treatment. The spatial distribution of strain and doping across the samples as a function of temperature is shown in Figures S9 and S10 for the treated flakes deposited on the 10 nm thick and 2 nm thick gold substrates, respectively. These images indicate that the variations occur homogeneously across both the inner part of the flake and its edges.

**Figure 4:**
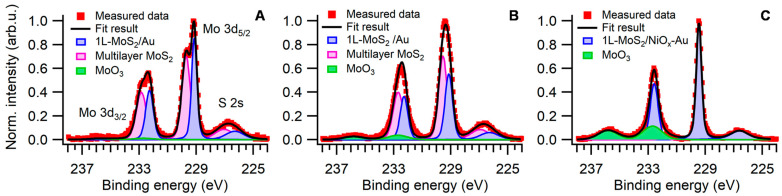
High-resolution Mo 3d XPS spectra of 1L-MoS_2_ flakes exfoliated on a 10 nm thick Au substrate. (**A**) XPS spectrum of the pristine sample, (**B**) after a 2 h thermal treatment at 225 °C under a 2 bar O_2_ atmosphere, and (**C**) after the same treatment at 300 °C (right).

To better understand the mechanisms leading to the increase in strain and decrease in concentration of negative charge carriers, XPS measurements were performed. Figure 4 shows the Mo 3d XPS spectra of MoS_2_ flakes on a 10 nm thick gold substrate (the spectra for MoS_2_ on a 2 nm thick Au are shown in the Supplementary Materials, Figure S11). The spectra were decomposed into two principal components with Mo 3d_5/2_ peaks centered at binding energies of 229.2 eV and 229.6 eV, respectively. The lower binding energy (BE) component is assigned to 1L-MoS_2_/Au, while the latter is attributed to two- and multi-layer MoS_2_ residues [40,41]. We propose that regions of 1L-MoS_2_ flakes not in close contact with the Au substrate, due to surface micro-roughness, also contribute to the latter component. A third component, corresponding to MoO_3_, appears on the higher binding energy side. The MoO_3_ signal is negligible in both the pristine sample (Figure 4A) and the sample treated at 225 °C (Figure 4B). In fact, in the pristine samples, the integrated intensity accounts for 3 ± 1% and 8 ± 2% of the total signal (3 ± 2% and 8 ± 3% for samples on a 2 nm Au substrate). Nonetheless, after thermal treatment at 300 °C, the MoO_3_ contribution increases to 29 ± 3% of the total signal. Moreover, the high-temperature treatment induced Au-Ni intermixing, as evidenced by the absence of a clear interface between the two materials in the HRTEM images (Figure S6C). The diffusion of Ni toward the surface was confirmed by an increased Ni 2p XPS core level signal of nickel and nickel oxides, which formed on top of the Au substrate (Figure S12). The altered chemical composition of the surface modified the MoS_2_ interaction with the substrate, shifting the principal Mo 3d component by 0.3 eV to higher BE (Figure 4C). No nickel surface segregation was observed in samples treated at 225 °C. These results suggest that, at 225 °C, the thermal conditions facilitate the incorporation of gaseous O_2_ to fill the *V*_S_ inherently present in the 1L-MoS_2_ structure; at the same time, at this temperature, minimal MoO_3_ formation occurs, thus preserving the structural integrity of the flakes. Based on these observations, a temperature of 225 °C was selected for all subsequent treatments, as this moderate temperature enables *V*_S_ filling while maintaining the sample integrity.

**Figure 5:**
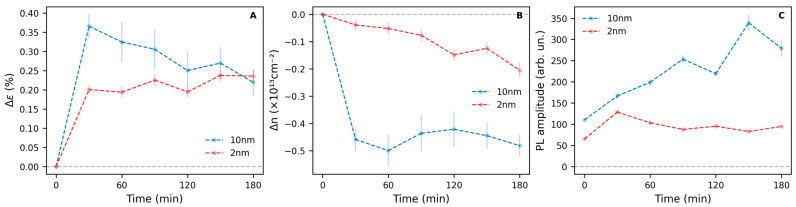
Strain variation Δε (**A**), negative charge-carrier concentration variation Δn (**B**), and cumulative PL amplitude (**C**) as a function of thermal treatment duration of 1L-MoS_2_ flakes exfoliated on two different Au substrates of thickness equal to 2 nm and 10 nm.

We thus investigated the kinetics of the thermal treatment performed at 225 °C. In particular, 30 min treatments were conducted at the selected temperature and subsequently repeated on the same sample, up to a total treatment duration of 3 h. As shown in Figure 5A, an increase in tensile strain is observed in the flakes on both substrates after the initial 30 min of treatment. Extending the treatment duration beyond this point does not induce significant further changes in strain. Regarding the concentration of the negative charge carrier, shown in Figure 5B, the flake deposited on the 10 nm Au layer exhibits a substantial decrease after the initial 30 min of treatment. However, for the flake on the thinner 2 nm gold substrate, n-type doping decreases gradually with increasing thermal treatment duration. Variations in strain and n-type doping in the flakes deposited on the 10 nm and 2 nm thick gold substrates, as shown in Figures S13 and S14, respectively, appear to proceed homogeneously across the surface of the samples. Compared to the treatments performed at increasing temperatures, the E’ bandwidth does not increase significantly (Figure S15), indicating that prolonged thermal treatments do not induce structural degradation in the samples.

In addition, as shown in Figure 5C, the intensity of the PL exhibited by 1L-MoS_2_ flakes increases after the thermal treatments. Interestingly, in the case of the flake deposited on the thicker 10 nm gold substrate, such an increase occurs progressively as the treatment duration is increased, while the maximum increase in the PL amplitude of the flake on the thinner 2 nm Au layer is observed already after the first 30 min. At the same time, no clear trend in the energy of the PL can be identified; in fact, although shifts in the position of the PL bands can be observed for some representative spectra (Figure S16A), such variations primarily occur within the distribution of PL energies for the 10 nm sample and with just one initial blueshift after 30 min for the 2 nm one (Figure S16B).

**Figure 6:**
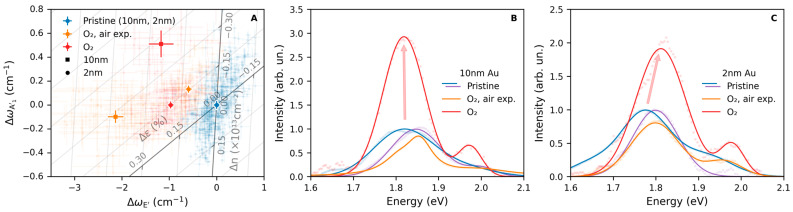
Map of strain and doping variation with respect to pristine samples (**A**) and PL spectra (**B**,**C**) of 1L-MoS_2_ flakes exfoliated on two different Au substrates of thickness equal to either 10 nm (**B**) or 2 nm (**C**) before and after a thermal treatment. After the treatments, the samples were either allowed to cool down naturally or by exposing them to the ambient air prior to characterization, as indicated by the legend. The strain-doping map in panel (**A**) displays points calculated across the surface of the studied flakes as partially transparent, while their average is shown as a solid color. The data points in panels (**B**,**C**) represent the experimental data, and the continuous lines represent the fits with Gaussian bands.

Finally, in order to understand the influence of the ambient environment on the overall variations observed in the properties of the 1L-MoS_2_ flakes, a 2 h treatment at 225 °C was repeated. The treatment was again carried out under a 2 bar O_2_ atmosphere; however, this time, at the end of the thermal process and before any characterization, the flakes were exposed to the ambient atmosphere to allow them to cool down to room temperature faster. After this treatment, as shown in Figure 6A, an increase in strain was again observed, accompanied by a decrease in n-doping compared to the pristine flakes. Considering only samples deposited on Au of the same thickness, the treatments carried out without any exposure to the ambient atmosphere result in variations in the negative charge-carrier concentration comparable to those observed under air exposure. At the same time, the increase in tensile strain of the flake deposited on a 10 nm thick Au substrate and exposed to the ambient is higher compared to the treatments carried out without exposure to air. In contrast, changes in strain of the flakes deposited on the thinner 2 nm Au layer occur in the opposite way, being slightly larger in the sample unexposed to the ambient environment.

Considering the PL exhibited by 1L-MoS_2_, as shown in Figure 6B,C, it can be observed that there is no increase in intensity when the samples are exposed to the ambient atmosphere while still at a high temperature. On the other hand, as previously observed, the PL of the samples increases when the flakes are not exposed to air. Additionally, in the case of the sample deposited on a 2 nm Au substrate, a blueshift of the A band equal to 0.05 eV was observed after the thermal treatment carried out without air exposure. Such a shift was not found in the other samples. These results suggest that the variations in tensile strain and negative charge carrier concentration are not the main factors for the observed increase in the flakes’ PL intensity. If this were the case, similar variations in strain and doping occurring in the flakes after thermal treatment would otherwise result in a comparable enhancement of the PL. The average variations in strain, doping, and relative PL amplitude compared to the pristine flakes after the thermal treatments are presented for all flakes in Table 1 for both thermal processes carried out with and without exposure to the ambient environment.

## 4. Discussion

The initial characterization of the pristine samples (Figure 1) shows that the gold-assisted mechanical exfoliation of MoS_2_ allows us to obtain 1L-MoS_2_ flakes with n-type doping and tensile strain. It is not unexpected that 1L-MoS_2_ flakes present n-type doping due to the presence of inherent *V*_S_ [25,26,32]. At the same time, the interaction with the underlying substrate is known to introduce tensile strain in 1L-MoS_2_ flakes [42]. The presented results further indicate that the characteristics of the underlying substrate can influence the properties of the exfoliated flakes. When the exfoliation was performed on the 10 nm thick gold substrate, the 1L-MoS_2_ obtained was similar to a hypothetical free-standing sample; on the other hand, flakes found on the thinner 2 nm gold layer exhibited greater tensile strain and a higher negative charge concentration. This is likely due to inhomogeneities in the substrate morphology, particularly relevant in the case of the thinner substrate. Indeed, the thicker 10 nm gold substrate had a roughness equal to 0.16 nm, while the thinner 2 nm substrate had a roughness of 0.5 nm, as measured by AFM. This evidence indicates that the deposition on such a thin gold layer can lead to inhomogeneities in the surface flatness, which then influence the properties of 1L-MoS_2_. Such a consideration becomes particularly important when considering the PL of the studied flakes, which is more intense in samples deposited on the thicker, higher quality 10 nm Au substrate. The enhanced PL exhibited by pristine flakes deposited on such substrate is probably due to the electronic coupling between the 1L-MoS_2_ and the Au substrate. The higher negative charge-carrier concentration observed in pristine flakes deposited on the 2 nm Au substrate suggests a stronger electronic coupling between the 1L-MoS_2_ and this substrate. Consequently, as reported in the literature, the higher n-type doping would lead to partial PL quenching [16,23].

Nonetheless, we have demonstrated that the properties of 1L-MoS_2_ can be controlled by carefully tailored thermal treatments. Our study shows that, even at mild temperatures, as low as 225 °C, it is possible to modify the material significantly. When, however, the same treatments are performed at high temperatures, considerable structural damage can be caused to the treated samples. This finding is rather unexpected given that bulk MoS_2_ is considered stable up to very high temperatures [43] and that 1L-MoS_2_ is produced via chemical vapor deposition at 650 °C or higher [31,42]. Our results, clearly evidenced through AFM and HRTEM observations, indicate that a high degree of strain builds up in the sample at 300 °C (Figure 3B), which may be the underlying cause of the witnessed structural damage (Figure 2C and Figure S4). The presence of MoO_3_, as detected by XPS, indicates that this damage may be caused by molybdenum oxidation (Figure 4 and Figure S10). The formation of this compound within the 1L-MoS_2_ structure is likely to induce an increase in the material strain, possibly due to the coexistence of different crystalline structures within the same flake, ultimately leading to the observed fractures.

On the other hand, when the treatment process is carried out under mild conditions, 1L-MoS_2_ damage can be avoided. AFM and HRTEM measurements show that, at 225 °C, the monolayer flake structure is preserved, while XPS characterization indicates a low MoO_3_ content in the samples following treatments performed at this temperature. These results are in line with the observed increase in strain after the treatments, calculated from the shift in Raman band positions compared to those of pristine samples. We hypothesize that the tensile strain of the material increases because of the mismatch between the thermal expansion coefficient (TEC) of the substrate and that of the 1L-MoS_2_ flake deposited on top of it. Gold exhibits a TEC of 14 × 10⁻^6^ K⁻^1^ [44], whereas 1L-MoS_2_ has been reported to have a TEC of approximately 7 × 10⁻^6^ K⁻^1^ [45]. Since the TEC of gold is double that of 1L-MoS_2_, the expansion of the gold substrate during the heating phase of the thermal treatments influences the flake attached to its surface. As the 1L-MoS_2_ is bound to the Au, the free expansion of the substrate leads to an increase in the sample tensile strain. Upon cooling the samples back to room temperature, the flakes do not fully relax to their initial state, thus explaining the observed strain differences after the treatments.

Furthermore, the thermal treatments were demonstrated to be capable of causing a decrease in the samples’ negative charge-carrier concentration. The unintentional n-type doping of 1L-MoS_2_ has been associated with *V*_S_ inherently present in the structure of the flakes [25,26,32]. The observed decrease in the doping would thus be consequently linked to a decrease in the concentration of these vacancies. It has been reported that O_2_ can interact with MoS_2_ by filling its sulfur vacancies [41]. As such, we hypothesize that the decrease in doping observed after the thermal treatments is caused by such a mechanism. This conclusion is further corroborated by the Mo 3d XPS peak shift by 0.15 eV to lower binding energies after the treatments. Such a core-level shift can be attributed to the different environments experienced by Mo atoms after the *V*_S_ are filled with O atoms. Overall, these results indicate that O_2_ is capable of interacting with 1L-MoS_2_ already at 225 °C.

The temperature at which the thermal treatments are carried out appears to have a role in the processes involving O_2_; in fact, the formation of MoO_3_ in 1L-MoS_2_ is thermodynamically unfavored at low temperatures while spontaneously occurring at 300 °C (Figure 4 and Figure S10). When considering the kinetics of the process, it was found that most of the effects already occur in the first 30 min of treatment (Figure 5). This is not surprising in the case of strain increase which, being associated with the mismatch between the TEC of 1L-MoS_2_ and substrate, would not be influenced by time as long as the treatments last long enough for the hypothesized bond rearrangement to occur. Conversely, in the case of the flake deposited on the thinner 2 nm gold substrate, the decrease in negative charge-carrier concentration appears to proceed with the treatment duration. This suggests that the filling of *V*_S_ by O atoms may be limited by the kinetics of the process.

Alterations in the shape and intensity of 1L-MoS_2_ PL are frequently associated with variations in its strain and negative charge-carrier concentration. It is known that increases in strain can cause a shift in the PL band’s position as well as a decrease in its overall intensity [40]. At the same time, changes in negative charge-carrier concentration can alter the intensity of the neutral A^0^ exciton in relation to the intensity of the charged A⁻ trion [16]. These combined effects are likely present in our results as well. Nonetheless, we hypothesize that these mechanisms alone are not sufficient to explain the observed increase in PL amplitude. Indeed, if changes in strain and doping were the only causes underlying the increase in PL intensity, similar variations in these properties would influence the PL in a comparable manner. On the contrary, a set of thermally treated 1L-MoS_2_ samples exposed to the ambient atmosphere displayed no increase in PL, despite showing variations in strain and doping comparable to those of samples not exposed to the ambient atmosphere, which instead, showed a substantial PL intensity increase. Such an observation indicates that the alterations in the strain and doping of 1L-MoS_2_ flakes observed during the thermal treatments can only partially explain the variations witnessed in the PL intensity.

Species present on the surface of 1L-MoS_2_ flakes can influence its optical properties. It is known that the interaction between molecular species present in the atmosphere and the monolayers is able to quench the 1L-MoS_2_ PL through mechanisms such as energy or electron transfers [46,47]. It can be hypothesized that the molecular species naturally present in the atmosphere, either H_2_O or more complex molecules, are able to adhere to the surface of MoS_2_, thus quenching the monolayer’s PL. By cleaning the material surface, it is possible to reverse this process and restore the intense PL. When performing the thermal treatments, the observed increase in PL might thus be due to the removal of species adsorbed on the surface of the samples. On the other hand, if during the thermal treatment or just at its end, the sample is exposed to the ambient environment before cooling down, the available thermal energy may be sufficient to enable atmospheric contaminants to rapidly interact with the cleaned sample surface, thereby restoring the contamination.

We thus conclude that, although variations in strain and doping can affect the optical properties of the studied material, such a mechanism is not the only one affecting the PL properties during the thermal treatments. In fact, it appears that the interaction with external species is also able to affect the final PL properties. This consideration can explain how exposing the material to the ambient atmosphere after the thermal treatments inhibits any enhancement in the 1L-MoS_2_ PL intensity amplitude while not affecting the variations in the strain and doping of the material.

## 5. Conclusions

We have studied the properties of 1L-MoS_2_ exfoliated on gold and explored the possibility of controlling its characteristics through controlled thermal treatments.

The initial characterization revealed that 1L-MoS_2_ flakes on a thicker 10 nm substrate present a stronger PL compared to flakes on a thinner 2 nm substrate. These results are significant from the research point of view, as the described exfoliation procedure, though influenced by the substrate quality, can yield 1L-MoS_2_ samples with properties close to an unperturbed reference. Additionally, we have studied the effect of thermal treatments under O_2_ on the properties of 1L-MoS_2_ and identified the optimal conditions for tuning the characteristics of the studied flakes without causing their degradation. We demonstrated that 1L-MoS_2_ flakes degrade at relatively low temperatures, particularly below 300 °C. However, at lower temperatures, we were able to modify the electronic properties of 1L-MoS_2_ and enhance its PL in just 30 min of treatment. Additionally, the treated samples showed a trend toward p-type doping, an important feature for the development of multi-layer devices based on 1L-MoS_2_ with opposite doping characteristics. Notably, the PL amplitude of the studied samples doubled after the thermal procedure. Such enhancement is very promising for the development of sensing devices exploiting the emission properties of 1L-MoS_2_ and for optoelectronic applications in general.

Finally, the causes underlying the observed effects were studied. Our study highlighted the ability of oxygen to interact with the *V*_S_ inherently present in the monolayer flakes, filling these vacancies and reducing the concentration of negative charge carriers. Moreover, the observed PL enhancement in 1L-MoS_2_ was linked to the removal of molecular species present on the surface of the studied flakes. Impurities such as H_2_O and other small molecules present in the atmosphere can interact with the surface of non-encapsulated 1L-MoS_2_. At the same time, depending on the flakes’ production route, residues from the synthesis process can also quench the PL of 1L-MoS_2_. The developed thermal process effectively removes such contaminants while, at the same time, reducing the n-type doping character of 1L-MoS_2_. These results are promising for both research and applications, as they facilitate the enhancement of 1L-MoS_2_ properties through a step that is separated from its synthetic route.

## Figures and Tables

**Table 1:** Calculated variations induced by thermal treatment in strain, negative charge-carrier concentration, and relative PL amplitude of 1L-MoS_2_ flakes exfoliated on two different Au substrates of thickness equal to either 10 nm or 2 nm. The treatments were carried out for 2 h at 225 °C under a 2 bar O_2_ atmosphere after which the samples were left to cool down naturally either in O_2_ or by exposing them to the air of the ambient environment, as indicated. Numbers in parentheses correspond to the uncertainties associated with each quantity to the precision of its least significant digit.

Au Layer Thickness	Air Exposure	Strain (%)	Doping (×10^13^ cm^−2^)	Relative PL Amplitude
10 nm	No	0.25 (5)	−0.42 (6)	1.99 (5)
	Yes	0.43 (3)	−0.28 (3)	0.93 (5)
2 nm	No	0.195 (15)	−0.15 (2)	1.46 (6)
	Yes	0.120 (12)	−0.150 (16)	1.04 (2)

## Data Availability

The original contributions presented in this study are included in the article/Supplementary Materials. Further inquiries can be directed to the corresponding author.

## Supplementary Materials

The following supporting information can be downloaded at: https://www.mdpi.com/article/10.3390/nano15030160/s1, Figure S1: height profile and AFM morphology of a pristine 1L-MoS_2_ flake; Figure S2: Raman spectra of pristine 1L-MoS_2_ flakes; Figure S3: strain distribution, negative charge-carrier concentration distribution, and optical microscopy images of 1L-MoS_2_ flakes; Figure S4: energy of the A and B PL bands and cumulative PL amplitude of pristine 1L-MoS_2_ flakes; Figure S5: optical microscopy images of 1L-MoS_2_ flakes as a function of thermal treatment temperature; Figure S6: HRTEM images of a 1L-MoS_2_ flake exfoliated on a 10 nm thick Au substrate before and after a 2 hour-long thermal treatment carried out either at 225 °C or 300 °C under a 2 bar O_2_ atmosphere; Figure S7: AFM phase images of a 1L-MoS_2_ flake in pristine conditions and after thermal treatments; Figure S8: FWHM of the E’ Raman band of 1L-MoS_2_ flakes as a function of thermal treatment temperature; Figures S9 and S10: strain and negative charge-carrier concentration distributions of 1L-MoS_2_ flakes as a function of thermal treatment temperature; Figure S11: High resolution Mo 3d XPS spectra of pristine and thermally treated 1L-MoS_2_ flakes on a 2 nm thick Au substrate; Figure S12: Ni 2p XPS spectra of pristine and thermally treated 1L-MoS_2_ flakes on a 10 nm thick Au substrate; Figures S13 and S14: strain and negative charge-carrier concentration distributions of 1L-MoS_2_ flakes as a function of thermal treatment duration; Figure S15: Raman spectra and FWHM of the E’ Raman band of 1L-MoS_2_ flakes as a function of thermal treatment duration; Figure S16: PL spectra and average emission energy of 1L-MoS_2_ flakes as a function of thermal treatment duration.
