# Supplementary material for: Mild Temperature Thermal Treatments of Gold-Exfoliated Monolayer MoS2
Source: Nanomaterials (Basel). 2025 Jan 22;15(3):160. doi: 10.3390/nano15030160 (PMC11820908; doi:10.3390/nano15030160)
Supplement: Supplementary file 1 [file nanomaterials-15-00160-s001.zip › nanomaterials-3426548-supplementary.pdf]

Mild Temperature Thermal Treatments of Gold-Exfoliated Monolayer MoS_2

Emanuele Sangiorgi [1], Antonino Madonia [1,*], Gianmarco Laurella [1], Salvatore Ethan Panasci [2], Emanuela Schilirò [2], Filippo Giannazzo [2], Igor Pis [3], Federica Bondino [3], György Zoltán Radnóczi [4], Viktória Kovács-Kis [4], Béla Pécz [4], Gianpiero Buscarino [1], Franco Mario Gelardi [1], Marco Cannas [1] and Simonpietro Agnello [1,5]

1 Department of Physics and Chemistry Emilio Segrè, University of Palermo, via Archirafi 36, I-90123 Palermo, Italy
2 CNR-IMM, Strada VIII n.5, Zona Industriale, I-95121, Catania, Italy
3 CNR - Istituto Officina dei Materiali (IOM), Area Science Park, S.S. 14 Km. 163, 5, Basovizza, I-34149 Trieste, Italy
4 HUN-REN Centre for Energy Research, Institute of Technical Physics and Materials Science, Konkoly-Thege ut 29-33, 1121 Budapest, Hungary
5 ATEN Center, University of Palermo, Viale delle Scienze Ed. 18, I-90128 Palermo, Italy
* Correspondence: antonino.madonia@unipa.it

Strain and doping values reported in the manuscript were calculated from the E′ and A_1′ phonon peaks positions of the 1L-MoS_2 Raman spectrum [S1]. The phonon frequencies $\omega^{E'}$ and $\omega^{A_1'}$ were evaluated through a least-squares fitting procedure using Lorentzian bands as model for each observed peak. The phonon frequency shift induced by strain and doping is described by the following expression:

$$\omega^m = \omega_0^m - 2\gamma^m \omega_0^m \varepsilon + k^m n \tag{1}$$

where, for each phonon mode m (E′ and A_1), ω_0^m is the unperturbed phonon peak frequencies, γ^m is the corresponding Grüneisen parameter for bi-axial strain, k^m is the shift-rate with charge-carrier concentration, and ε and n are the strain and negative charge-carrier concentration respectively.

From the linear combination of the equation (1) expressed for the E′ and A_1′ phonon modes it is possible to calculate the strain and negative charge-carrier concentration of the material as follows:

$$\varepsilon = \frac{k^{A'_1{}'}\left(\omega^{E'} - \omega_0^{E'}\right) - k^{E'}\left(\omega^{A_1'} - \omega_0^{A_1'}\right)}{2\gamma^{A_1'}\omega_0^{A_1'}k^{E'} - 2\gamma^{E'}\omega_0^{E'}k^{A_1'}} \tag{2}$$

$$n = \frac{\gamma^{A_1'}\omega_0^{A_1'}\left(\omega^{E'} - \omega_0^{E'}\right) - \gamma^{E'}\omega_0^{E'}\left(\omega^{A_1'} - \omega_0^{A_1'}\right)}{\gamma^{A_1'}\omega_0^{A_1'}k^{E'} - \gamma^{E'}\omega_0^{E'}k^{A_1'}} \tag{3}$$

Here the unperturbed phonon frequencies $\omega_0^{E'}$ = 385 cm^{-1}, $\omega_0^{A_1'}$ = 405 cm^{-1} were chosen based on the literature on suspended 1L-MoS_2; values $\gamma^{E'} = 0.68$ and $\gamma^{A_1'} = 0.21$ were used as bi-axial strain Grüneisen parameter while values $k^{E'} = -0.33 \times 10^{-13}$ cm^{-1} and $k^{A_1'} = -2.22 \times 10^{-13}$ cm^{-1} were used for the shift-rate with charge-carrier concentration [S2]. The reported set of equations represent the linear transformations between the vector spaces $\left(\omega^{E'}, \omega^{A_1'}\right)$ and (ε, n) which has been used to construct the strain-doping maps presented in the article.

S1. Michail, A.; Delikoukos, N.; Parthenios, J.; Galiotis, C.; Papagelis, K. Optical Detection of Strain and Doping Inhomogeneities in Single Layer MoS_2. *Appl. Phys. Lett.* **2016**, *108*, 173102, doi:10.1063/1.4948357.

S2. Panasci, S.E.; Schilirò, E.; Greco, G.; Cannas, M.; Gelardi, F.M.; Agnello, S.; Roccaforte, F.; Giannazzo, F. Strain, Doping, and Electronic Transport of Large Area Monolayer MoS_2 Exfoliated on Gold and Transferred to an Insulating Substrate. *ACS Appl. Mater. Interfaces* **2021**, *13*, 31248–31259, doi:10.1021/acsami.1c05185.

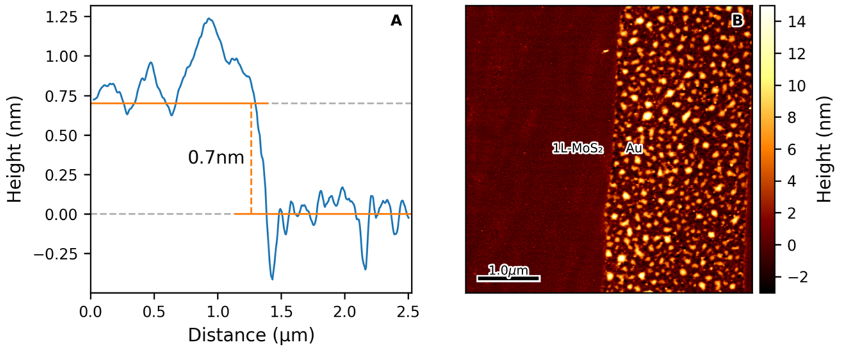

Figure S1. Height profile (**A**) and 5 × 5 μm² AFM morphology (**B**) of a pristine 1L-MoS_2 flake exfoliated on a 10 nm thick Au substrate. Regions associated to 1L-MoS_2 and Au have been labeled accordingly.

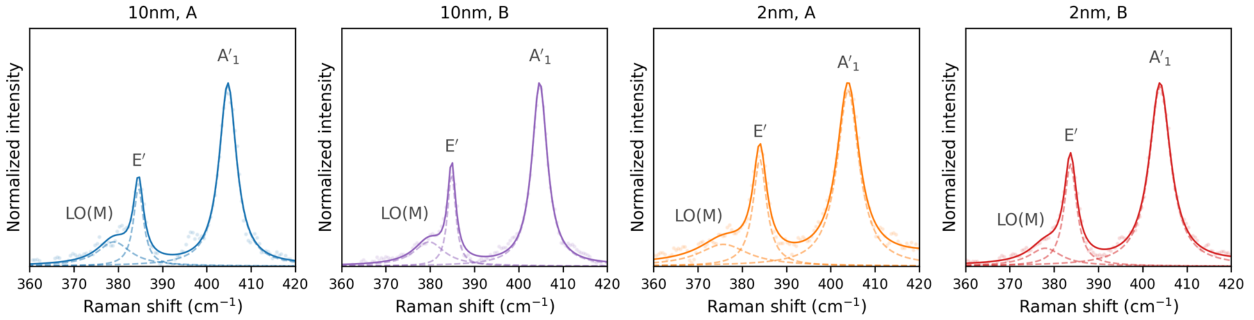

Figure S2. Raman spectra recorded at two different positions of pristine 1L-MoS_2 flakes exfoliated on two different Au substrates of thickness equal to either 2 nm or 10 nm. In the panels, points represent experimental data, continuous lines their fitting with Lorentzian bands, and dashed lines the individual fitting components which have been accordingly labeled.

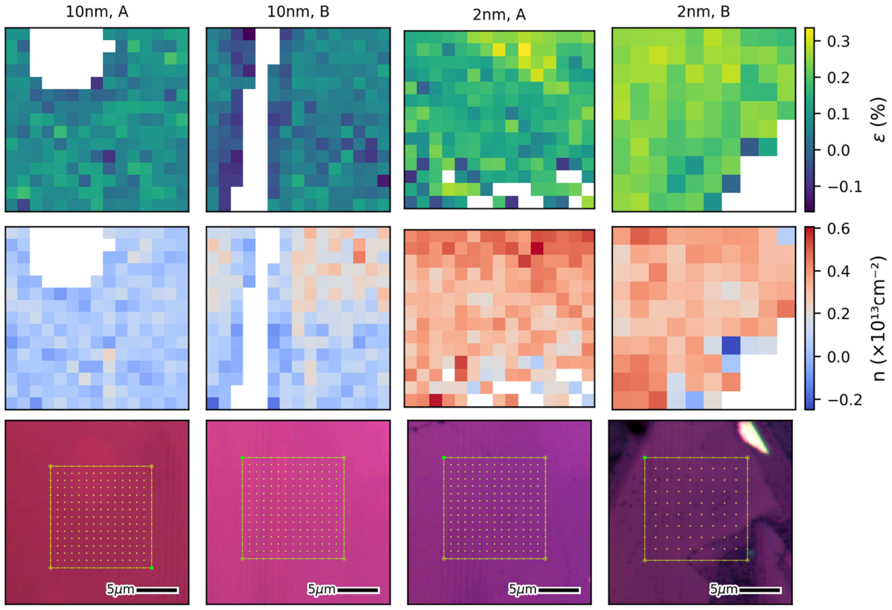

Figure S3. Strain distribution (**top**), negative charge-carrier concentration distribution (**middle**), and optical microscopy images (**bottom**) of pristine 1L-MoS_2 flakes exfoliated on a 10 nm or 2 nm thick Au substrate. Strain and doping distributions have been calculated from Raman spectra collected over a 15 × 15 μm² area in two different positions for each sample.

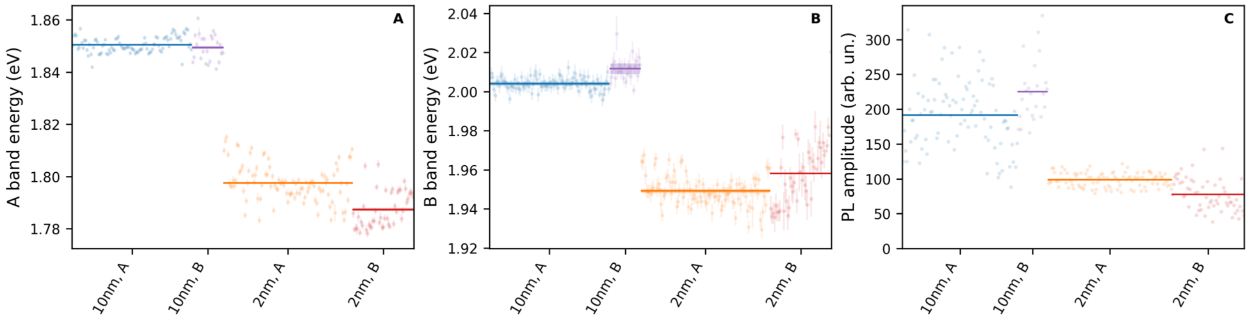

Figure S4. Energy of the A and B PL bands (panels **A** and **B**) and cumulative PL amplitude (panel **C**) of pristine 1L-MoS_2 flakes exfoliated on a 10 nm or 2 nm thick Au substrate. The average of each dataset is shown as a horizontal line.

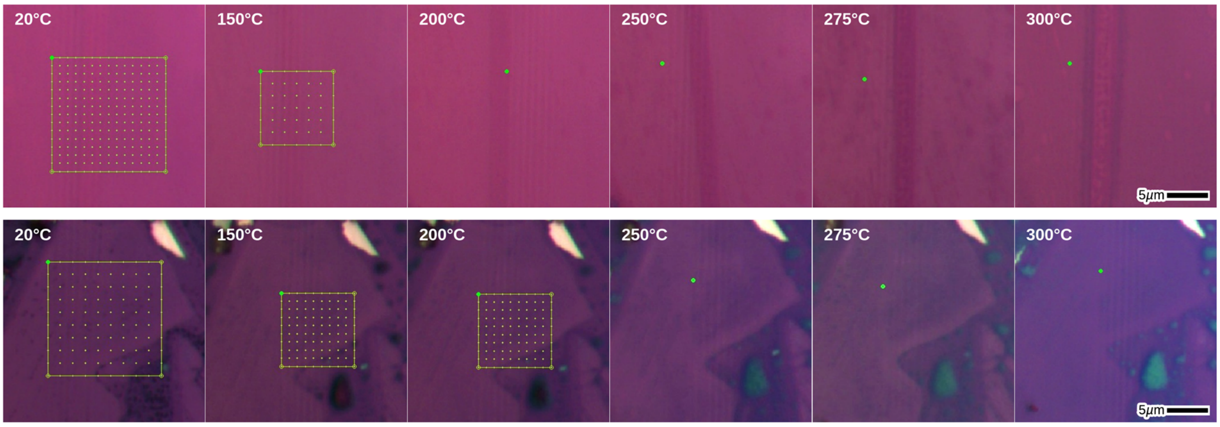

Figure S5. Optical microscopy images 1L-MoS_2 flakes exfoliated on a 10 nm (**top**) or 2 nm (**bottom**) thick Au substrate acquired after performing a 2 hour-long thermal treatment under a 2 bar O_2 atmosphere. The temperature at which the treatment was carried out is shown in the top-left corner of each image.

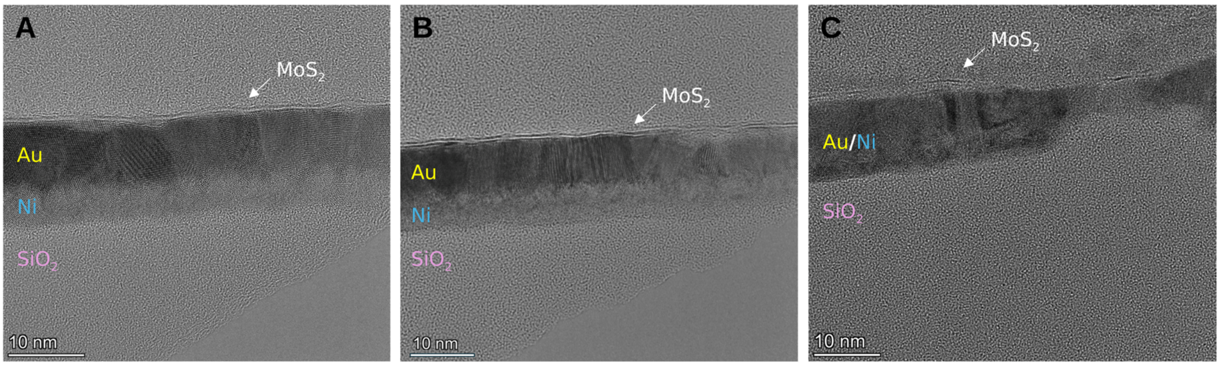

Figure S6. HRTEM images of a 1L-MoS_2 flake exfoliated on a 10 nm thick Au substrate. The panels show the morphology of a sample in pristine conditions (**A**), after a 2 hour-long thermal treatment carried out at 225 °C under a 2 bar O_2 atmosphere (**B**), and after the same treatment was carried out at 300 °C (**C**). Regions associated to 1L-MoS_2 and substrate have been labeled accordingly.

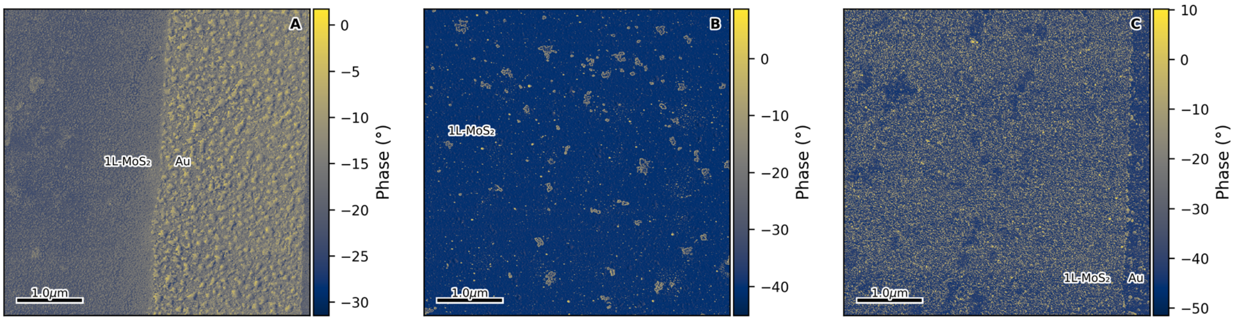

Figure S7. 5 × 5 μm2 AFM phase of a 1L-MoS_2 flake exfoliated on a 10 nm thick Au substrate. The panels show the phase of a sample in pristine conditions (**A**), after a 2 hour-long thermal treatment carried out at 225 °C under a 2 bar O_2 atmosphere (**B**), and after the same treatment was carried out at 300 °C (**C**). Regions associated to 1L-MoS_2 and Au have been labeled accordingly.

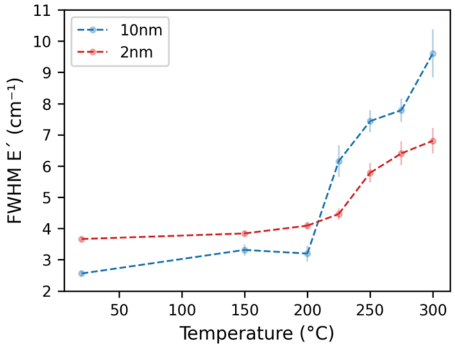

Figure S8. FWHM of the E´ Raman band of 1L-MoS_2 flakes exfoliated on two different Au substrates of thickness equal to either 2 nm or 10 nm as a function of thermal treatment temperature. Thermal treatments were carried out for 2 hour under a 2 bar O_2 atmosphere. In order to calculate the reported error bars, three times the standard error obtained from the fit of the Raman peaks has been used.

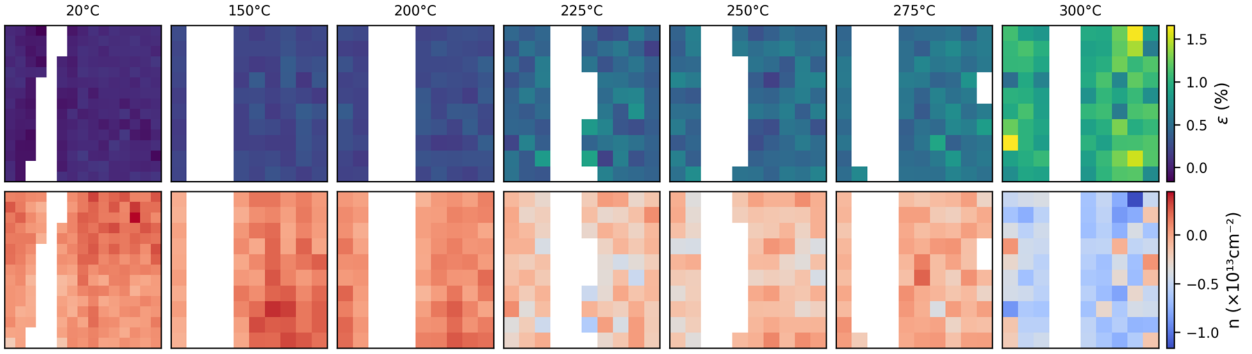

Figure S9. Strain (**top**) and negative charge-carrier concentration (**bottom**) distributions of a 1L-MoS_2 flake exfoliated on a 10 nm thick Au substrate as a function of thermal treatment temperature. Thermal treatments were carried out for 2 hour under a 2 bars O_2 atmosphere. The shown distributions have been calculated from Raman spectra collected over a 10 × 10 μm² area except for the 20 °C datasets which cover a 15 × 15 μm² area.

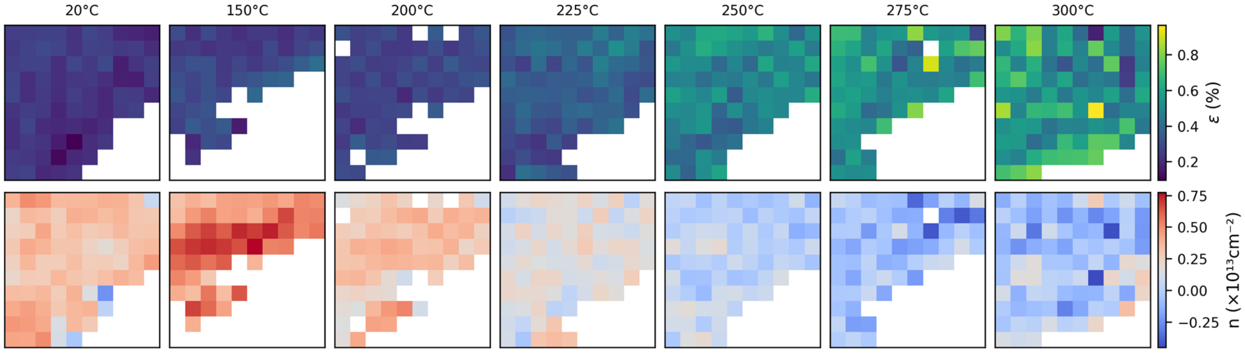

Figure S10. Strain (**top**) and negative charge-carrier concentration (**bottom**) distributions of a 1L-MoS_2 flake exfoliated on a 2 nm thick Au substrate as a function of thermal treatment temperature. Thermal treatments were carried out for 2 hour under a 2 bar O_2 atmosphere. The shown distributions have been calculated from Raman spectra collected over a 10 × 10μm² area except for the 20 °C datasets which cover a 15 × 15 μm² area.

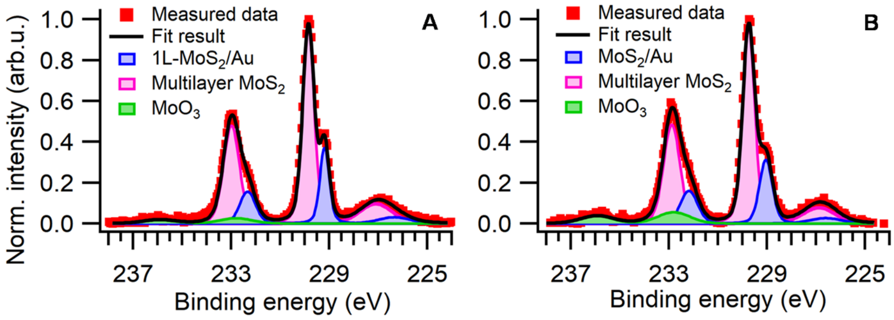

Figure S11. High-resolution Mo 3d XPS spectra of a 1L-MoS_2 flake exfoliated on a 2 nm thick Au substrate. (A) XPS spectrum of the pristine sample and (B) after a 2 hour treatment at 225 °C under a 2 bar O_2 atmosphere.

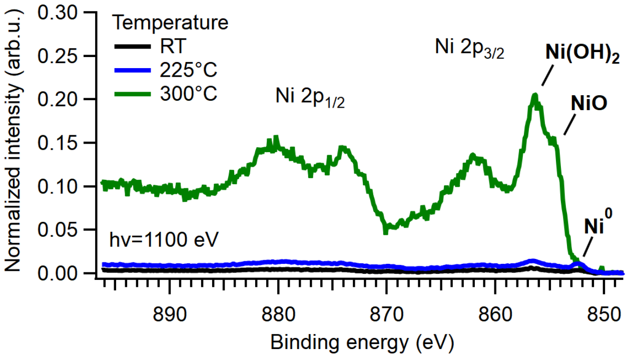

Figure S12. Ni 2p XPS spectra of 1L-MoS_2 flakes exfoliated on a 10 nm thick Au substrate after 2 bar O_2 treatment at the indicated temperatures. The intensities are normalized to the intensity of the Au 4f signal.

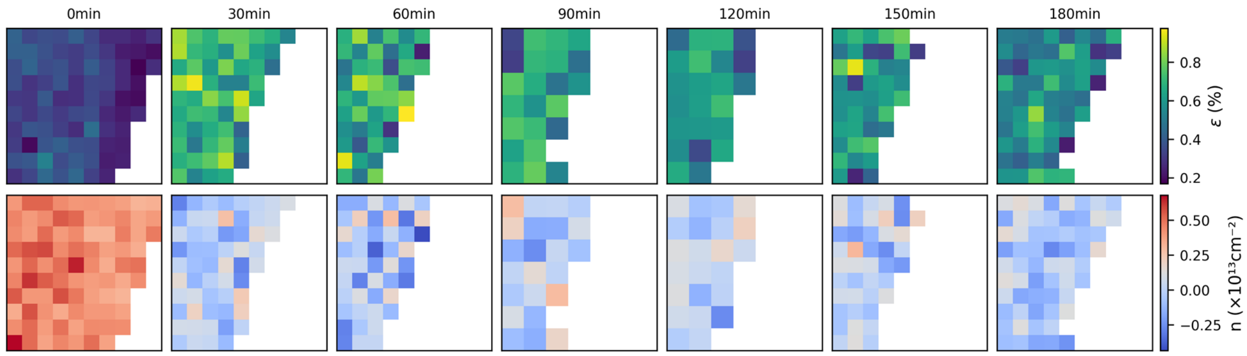

Figure S13. Strain (**top**) and negative charge-carrier concentration (**bottom**) distributions of a 1L-MoS_2 flake exfoliated on a 10 nm thick Au substrate as a function of thermal treatment duration. Thermal treatments were carried out at 225 °C under a 2 bar O_2 atmosphere. The shown distributions have been calculated from Raman spectra collected over a 10 × 10 μm² area.

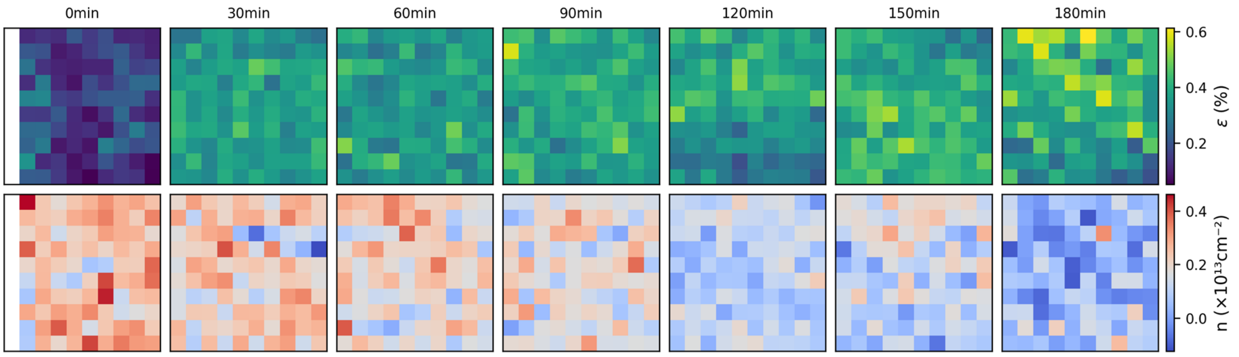

Figure S14. Strain (**top**) and negative charge-carrier concentration (**bottom**) distributions of a 1L-MoS_2 flake exfoliated on a 2 nm thick Au substrate as a function of thermal treatment duration. Thermal treatments were carried out at 225 °C

under a 2 bar O_2 atmosphere. The shown distributions have been calculated from Raman spectra collected over a 10×10 μm² area.

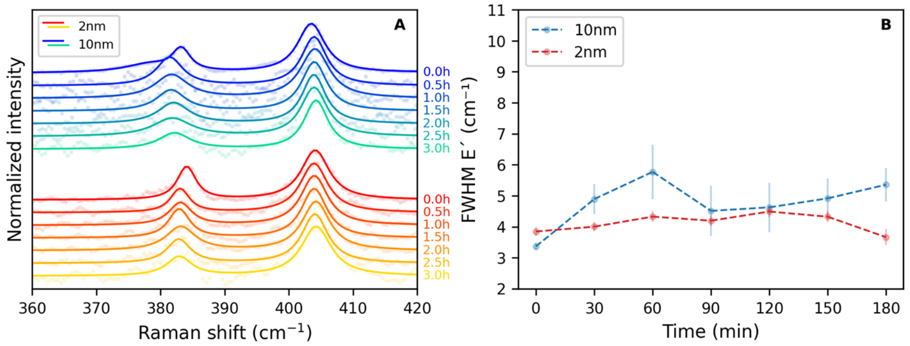

Figure S15. Raman spectra (**A**) and FWHM of the E´ Raman band (**B**) of 1L-MoS_2 flakes exfoliated on a 10 nm or 2 nm thick Au substrate as a function of thermal treatment duration. Thermal treatments were carried out at 225 °C under a 2 bar O_2 atmosphere. Raman spectra are fitted with three Lorentzian shaped peaks associated to the LO(M), E´ and A1´ modes. In order to calculate the reported error bars, three times the standard error obtained from the fit of the Raman peaks has been used.

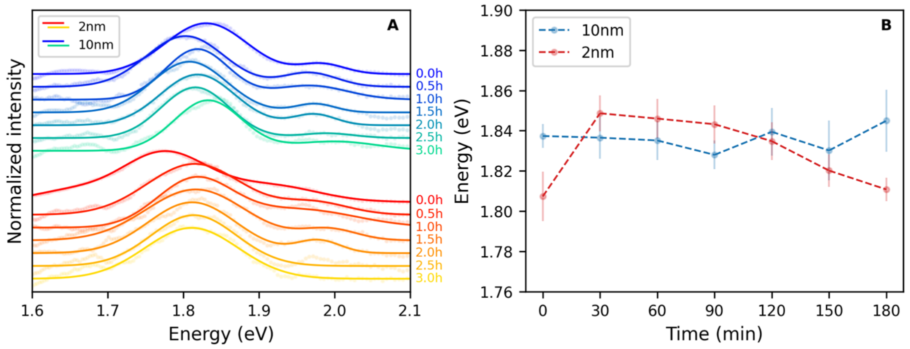

Figure S16. PL spectra (**A**) and average emission energy (**B**) of 1L-MoS_2 flakes exfoliated on a 10 nm or 2 nm thick Au substrate as a function of thermal treatment duration. Thermal treatments were carried out at 225 °C under a 2 bar O_2 atmosphere. The PL spectra are fitted with two Gaussian shaped bands associated to the A and B exciton emission. The reported error bars correspond to the standard deviation of each PL energy distribution at a certain treatment duration.
